# Biomolecular Markers in Cancer of the Tongue

**DOI:** 10.1155/2009/412908

**Published:** 2009-08-19

**Authors:** Daris Ferrari, Carla Codecà, Jessica Fiore, Laura Moneghini, Silvano Bosari, Paolo Foa

**Affiliations:** Departments of Oncology and Pathology, San Paolo Hospital, University of Milan, 20142 Milano, Italy

## Abstract

The incidence of tongue cancer is increasing worldwide, and its aggressiveness remains high regardless of treatment. Genetic changes and the expression of abnormal proteins have been frequently reported in the case of head and neck cancers, but the little information that has been published concerning tongue tumours is often contradictory. This review will concentrate on the immunohistochemical expression of biomolecular markers and their relationships with clinical behaviour and prognosis. Most of these proteins are associated with nodal stage, tumour progression and metastases, but there is still controversy concerning their impact on disease-free and overall survival, and treatment response. More extensive clinical studies are needed to identify the patterns of molecular alterations and the most reliable predictors in order to develop tailored anti-tumour strategies based on the targeting of hypoxia markers, vascular and lymphangiogenic factors, epidermal growth factor receptors, intracytoplasmatic signalling and apoptosis.

## 1. Introduction

Oral cancer is the most frequent cancer affecting the cervicofacial district, causing about 8000 deaths every year in the United States [1, 2], and cancer of the tongue accounts for approximately 30% of all oral cancers. The most frequent histological type is squamous cell carcinoma (SCC) which mainly affects men in the sixth decade of life [3–6]. The incidence of tongue cancer increased from 1973 to 2001 at the same rhythm as tonsil cancer, and about 10, 000 new cases were recorded in the United States in 2007 [7]. According to the Scandinavian registries, the trend towards an increasing incidence only excludes women 65–79 years old [8].

Unlike other studies, survival analyses have demonstrated that survival rates are better among young adults than older patients [9–11], with a 5-year crude survival rate of 65% (95% CI 59–71%) against 45% (95% CI 43–48%) in subjects aged 40–64 years and 33% (95% CI 31–35%) in those aged 65–79 years. Base of the tongue cancer has a poorer prognosis than mobile tongue cancer; according to US National Cancer Database findings, the 5- and 10-year disease-specific survival (DSS) rates for base of the tongue tumours are, respectively, 40.3% and 29.4%, and overall survival (OS) rates are, respectively, 27.8% and 12.2%. An older age (>65 years), low economic income, and advanced stage are independently associated with lower DSS, which is 64.7% for stage I and 30.0% for stage IV [12].

Smoking and alcohol consumption are recognised risk factors for tongue cancer, but are frequently not involved in the case of younger patients [13, 14]. Head and neck cancer (HNC) is heralded by some changes in genetic and epigenetic patterns, with gene inactivation or amplification being the main alterations that can lead to derangements in the molecular pathways involved in regulating cell behaviour [15–23]. Al-Moustafa et al. [24] found that genes encoding for growth factors and cell structure were overexpressed in 0.7% of their cases, and those involved in cell motility and apoptosis were underexpressed in 1%: more specifically, at protein level, Wnt-5a, fibronectin and N-cadherin were upregulated, whereas E-cadherin, claudin-7, the catenins, and connexin 31.1 were downregulated. However, the specific relationships between genes and proteins, the final alteration that may imprint the neoplastic clone and its development, have not yet been ascertained.

Ongoing biological research is attempting to establish whether these proteins can be considered biomarkers that could guide therapeutic choices. SCC of the tongue is characterised by an unpredictable course as some patients with early lesions may develop local recurrence and regional metastases despite adequate surgery, and so the identification of prognostic markers would enable clinicians to target patients who may benefit from a specifically tailored treatment strategy. 

This review will concentrate on the most recent advances in the rapidly evolving field of biomarker research in this tumour type.

## 2. Viral Infections

Two viruses are commonly associated with HNC. The integration of Epstein-Barr virus (EBV) into mucosal cells is the most important pathogenetic factor in the development of nasopharyngeal carcinoma, which is endemic in geographical areas such as the Middle East and South-East Asia, the Arctic area, and Northern Africa [25–30]. EBV is transmitted through saliva, but its cell source is controversial, although putative reservoirs include the oral epithelium and salivary glands. Frangou et al. [31] observed EBV replication in 1.3% of tongue mucosal samples, but no latent infection was found, and EBV infection was not detected in the tongue carcinomas. It is, therefore, reasonable to argue that EBV replication occurs infrequently in tongue epithelial cells, and that EBV is probably not involved in the pathogenesis of tongue cancer.

Oropharyngeal cancer is closely associated with human papilloma virus (HPV), whose growing incidence in young adults accounts for a proportional increase in the incidence of tonsil cancer. Subtypes 16 and 18 are commonly involved in the pathogenesis of oropharyngeal carcinoma [32], and are suspected of increasing the risk of tongue cancer by 3–5 times [33–35]. The prevalence of HPV in tongue cancer varies considerably but, when it is present, the median copy numbers of E6 DNA in nontonsillar specimens is approximately 80, 000 times lower than in tonsillar specimens [36]. Kantola et al. [37] found that none of 105 mobile tongue cancer patients harboured HPV, and two studies have reported HPV frequencies in oral tongue cancer of 2.3% and 1.96%, thus confirming its small etiopathogenetic role, at least in the mobile portion of the tongue [38, 39]. Liang et al. [39] reported a higher incidence of HPV in base of the tongue cancer (51.5%), and Dahlgren et al. [38] stated that mobile and base of the tongue SCC are different diseases, with HPV being present in 40% of the patients affected by the latter and, as has been observed in the case of tonsillar cancer, the presence of HPV in base of the tongue cancer positively influenced survival (*P* = .0159). Interestingly, the HPV-positive base of the tongue cancer patients still had an advantage over those who were HPV-negative in terms of 5-year DSS (*P* = .0362), whereas tumour stage at the time of diagnosis no longer had an impact (*P* = .0863) [38]. The presence of HPV is, therefore, clearly associated with a better prognosis, and outweighs the predictive value of disease stage.

## 3. Hypoxia: Follow-Up (a)

Deranged vascular architecture and necrotic changes within neoplastic tissue are responsible for tumour hypoxia, which is associated with a poor outcome in HNC patients [40, 41]. Poorly oxygenated tumours have a poor prognosis as they may be resistant to radio- and chemotherapy, and favour malignant progression [42–45]. Tumour cells harbouring genetic alterations survive longer than normal cells in a hypoxic environment and are more likely to transmit genomic instability as a consequence of selective pressure, after which the neoplastic clone can easily grow, increase angiogenesis and motility, and finally spread through the lymphatic system or blood vessels [45–48].

It is thought that hypoxia upregulates some highly expressed proteins that are easily recognised immunohistochemically and may act as endogenous biomarkers in HNC [45]. Hypoxia-inducible factor 1a (HIF-1*α*) is a partner in a dimer that acts as a transcription factor by binding a specific DNA sequence and activating gene transcription. Under hypoxic conditions, HIF-1*α* levels increase and activate genes coding for growth and angiogenesis factors, as well as glycolytic enzymes. It has been demonstrated that such genes, particularly, carbonic anhydrase IX (CA-9), vascular endothelial growth factor (VEGF), and erythropoietin (EPO), are highly expressed in HNC [49, 50] but transferring immunohistochemical results to the clinical setting in order to identify their real prognostic value and impact on clinical practice is difficult.

Roh et al. [51], retrospectively, studied T2 tongue cancer using monoclonal antibodies against HIF-1*α*, HIF-2*α*, CA-9, the glucose transporter (GLUT-1) and EPO receptors (EPORs), and found that only GLUT-1 was related to nodal stage and could, therefore, be used as a potential predictor of nodal metastates. Univariate analysis showed that HIF-1*α* and EPOR expression significantly correlated with DSS (*P* < .05), but not with other clinicopathological variables such as tumour thickness, nodal involvement, and resection margin status, and multivariate analysis showed that only EPOR expression remained a significant predictor of DSS (*P* = .030). However, the small number of patients and the fact that they all had T2 tongue cancer makes it difficult to draw any definite conclusions.

The role of exogenous hypoxia markers is beyond the scope of this review, but it is worth mentioning the role of pimonidazole, a marker of exogenous hypoxia in human SCC of the cervix and head and neck [52–54]. The pimonidazole binding assay is a direct indicator of tumour hypoxia, which has been proved to be significantly associated with locoregional control and disease-free survival (DFS) [55]. Patients with hypoxic tumors show a worse initial response to treatment and have more locoregional recurrences during the first 15 months of followup, thus suggesting that their worse outcome mainly depends on early locoregional failures.

## 4. VEGF

Vascular endothelial growth factors (VEGFs) are a family of proteins with specific angiogenic properties that increase vessel permeability, and endothelial cell growth, proliferation, migration, and differentiation [56, 57]. VEGF-A/vascular permeability factor and VEGF-C have been recently recognised as lymphangiogenic/angiogenic factors that induce lymph and blood vessel hyperplasia and facilitate tumour progression and metastases [58, 59]. VEGF-A consists of four isoforms with a different molecular mass (121, 165, 189, and 206 amino acids) and different biological activity [60]. VEGF-C is structurally very similar to VEGF-D, and both of these and their major receptors (VEGFR-2 and VEGFR-3) are expressed in many cancer cells and may regulate lymphangiogenesis by facilitating the signalling network between endothelial and cancer cells [61–66]. A significant correlation has been demonstrated between VEGF-A and VEGF-C expression and lymph node metastases [67, 68], and patients overexpressing these two factors tend to show decreased survival. On the contrary, VEGF-D has been found to be underexpressed in HNC cells, and it is thought that it has an antagonistic effect on other VEGFs and may play a role in the late process of neoangiogenesis stabilisation.

Kishimoto et al. [69] investigated the association between VEGF-C expression and regional lymph node metastases in oral squamous cell carcinoma (OSCC) by examining its immunohistochemical expression in biopsy specimens obtained from 62 patients. In the early stages (T1 and T2), VEGF-C expression closely correlated with lymph node metastases (*P* < .001), but there was no significant correlation in the advanced stages (T3 and T4). These findings indicate that VEGF-C expression in biopsy specimens could be used as a reliable predictor of regional lymph node metastases, particularly in early OSCC, and may become an important factor when choosing the most appropriate treatment.

The limited data concerning tongue cancer are conflicting. Kim et al. studied the expression of VEGF and metalloproteinase-2 and -9 in 38 oral tongue cancer patients, and found a significant correlation between VEGF expression and the extent of tumour invasion (*P* = .002). Furthermore, the tumour-free survival of the VEGF-positive patients was significantly worse than that of the VEGF-negative patients (*P* = .019) [70]. However, Faustino et al. did not find a a similar correlation in early stage OSCC: 60 out of 87 patients (68.9%) were affected by tongue cancer and it was found that VEGF-C expression did not predict occult lymph node metastases in T1-T2N0 tumours [71]. In another study, Cho et al. found high VEGF expression in 20 out of 33 specimens of resected tongue cancer (60.6%), but no correlation between it and recurrence (*P* = .33) [72]; the expressions of maspin, an inhibitor of angiogenesis and tumour suppressant [73, 74], and mutant-type p53 were also evaluated but did not correlate with recurrent disease. We studied 56 patients undergoing radical surgery for tongue cancer and found that the expression of VEGF-C and its receptor VEGFR-2 correlated with DFS but not OS (unpublished data). Although there is some evidence that VEGF-C plays a role in causing more aggressive tongue cancer, further studies of larger patient series are needed.

## 5. Tight Junctional Proteins

There are three main types of intercellular junctions: tight, adherens, and gap junctions. The most apical components of the junctional complexes are tight junctions (TJs), which play a major role as paracellular barriers to the transport of ions, water, and proteins, and are also believed to be involved in the signalling cascades controlling cell growth and differentiation. Together with desmosomes, they form part of cell-to-cell adhesion apparatuses, and strongly regulate the invasion of cancer cells [75–81]. TJs are involved in the neoplastic process because they couple the extracellular milieu to intracellular signalling pathways and the cytoskeleton [82]. Deranged TJ permeability may increase the diffusion of nutrients and other factors that promote tumour growth and/or survival [83]. 

Claudins and occludin are tight junctional proteins whose expression has been studied in various tumour types [84], and tentatively correlated with tumour proliferation as a result of a mechanism involving the activity of matrix metalloproteinases [85]. Claudin 7 is known to be underexpressed in HNC [24], but only Bello et al. have described its expression in tongue cancer. They analysed the distribution of claudins (1, 4, 5, and 7) and occludin in 97 patients with superficial and invasive front of tongue cancer, and found that claudins 1 and 7 were strongly expressed, claudin 4 moderately expressed, and claudin 5 the least expressed; occludin staining was irrelevant. Cause-specific survival analysis showed that, in comparison with intermediate immunoreactivity, high and low claudin 7 immunoreactivity tended to be associated with decreased survival [86]. The authors suggested that claudin 7 levels could be used for prognostic purposes, but the subjective nature of the immunohistochemical evaluation requires caution.

## 6. ErbB2-Ki-67: Tyrosine-Kinase (b)

ErbB2 (HER-2/neu) is a tyrosine kinase transmembrane receptor that belongs to the family of epidermal growth factor receptors (EGFRs), like ErbB1/HER-1, ErbB3/HER-3, and ErbB4/HER-4. It can be activated by means of heterodimerisation with the other members of the family, and is involved in cell proliferation and differentiation. It also plays a major role in tumour invasion via mitogen-activated protein kinase (MAPK) or phosphatidylinositol 3-kinase-(PI3K) AKT-activated pathways [87, 88]. Many (but not all) authors have demonstrated ErbB2 overexpression and gene amplification in oral SCC, and that they are associated with early recurrence, local, and distant metastases, or shorter survival [89–94]. Fatty acid synthase (FAS) is located in the cytosol and is responsible for the endogenous synthesis of saturated long-chain fatty acids. Its expression is upregulated in a number of human epithelial malignancies, including OSCC [95–100]. It has been shown that the overexpression of human ErbB2 in mouse fibroblasts stimulates FAS protein expression through a PI3K-dependent pathway. FAS is essential for cell proliferation, and its specific inhibition reduces cell growth, blocks DNA replication, and promotes apoptosis in various cancer cell lines [101, 102]. 

Silva et al. have shown that intracytoplasmatic ErbB2 expression correlates with the 10-year survival of tongue cancer patients, which was 24.1% in the case of high expression, and 53.4% in the case of weak or negative expression (log-rank test, *P* = .0096). The proliferation index, evaluated by means of Ki-67, significantly predicted both OS (log-rank test, *P* = .0001) and DFS (log-rank test, *P* = .0047); however, it did not correlate with the cell surface coexpression of FAS and ErbB2, thus indicating a favourable prognosis in both cases [103]. The same group has also studied the microscopic characteristics of tongue cancer, and found that histological grade (*P* < .05), lymphatic permeation (*P* < .001), perineural infiltration (*P* < .05), and nodal metastases (*P* < .02) are all associated with FAS status. High FAS expression correlates with aggressive histological features and may be important for tumour progression [104]. Different results have been obtained in tongue cancer using the Hercept test, which demonstrated that the expression of Erb B2 does not correlate with clinicopathological parameters and is not useful in treatment decision making [105].

## 7. p-53

The p53 tumour suppressor gene is located on the short arm of human chromosome 17 and encodes for a phosphoprotein that has dual activity on normal cells: it inhibits cell proliferation by arresting it at the G1-phase after DNA damage, and it induces apoptosis after genotoxic damage. It is thought that both mechanisms also suppress tumour growth [106].

The expression of p53 is strikingly important in the response to irradiation or cytotoxic drugs, and it has been shown that an alteration in the p53 gene may cause treatment failure in cancer patients as it prevents the triggering of the apoptotic pathway [107, 108]. Many environmental factors can alter p53 function, as has been demonstrated in the case of cigarette smoking and asbestos exposure in lung cancer [109, 110]. Exogenous factors can easily and directly act on cells in the oral cavity, but the role of cigarette smoking in deregulating p53 protein is still unclear [111, 112]. 

Atula et al. [113] have studied p53 mutations and protein expression in tongue cancer, and found mutations in 54% of the samples by means of single-stranded conformation polymorphism (SSCP) analysis, which correlated with tumour size (41% in T1-2 versus 90% in T3-4; Fisher's exact test, *P* < .01) and grading (75% of grade 2-3 versus 32% of low-grade cancers; chi-squared test, *P* < .01). Although experimental models have demonstrated that p53 mutations precede and favour the appearance of metastases [114, 115], this study found no correlation between metastases and p53 mutations or protein expression, a finding that can be explained on the grounds of the progressive accumulation of mutations during the course of cancer or viewed as an early event contributing to more aggressive behaviour. Tongue cancer develops as a sum of several environmental and genetic factors affecting the same cell, thus leading to its progressive malignant transformation and metastatic dissemination [116]. 

The expression of p53 in oral leukoplakia is higher than in cancer of the tongue and should probably be considered an early event in tumour progression [117]. Nagler et al. studied 116 patients with tongue cancer, and found the 5-year probability of OS was 55%, and better for mobile tongue than base of the tongue cancer (70% versus 32%, *P* = .0008). Immunohistological analysis of p53, the antiapoptotic protein Bcl-2 and c-erbB-2, and an assessment of the rate of apoptosis by means of terminal dUTP nick-end-labelling (TUNEL), in 55 specimens, revealed a significant correlation between p53 and TUNEL staining, but the link with prognosis needs to be studied further [118]. It has also been found that p53 positivity is not a reliable means of selecting patients for elective neck dissection in the management of N0 oral tongue cancer [119].

## 8. Osteopontin

Osteopontin (OPN) is a calcium binding protein that binds alpha rather than beta integrin and CD44 receptors, and activates intracellular signalling pathways associated with cell adhesion and migration [120–122]. It is expressed and secreted by many kinds of cancers, and has been associated with tumour progression and invasion [123–126]. It can also be induced by VEGF and is involved in vessel angiogenesis and endothelial cell survival [127–129]. Matsuzaki et al. [130] failed to demonstrate a correlation between OPN expression and lymphatic metastases and survival in T1-4 tongue cancer, but OPN expression has also been studied in T1-2 tongue cancer using a different means of immunohistochemical evaluation [131]. Thirty out of 94 patients (31.9%) expressed OPN and this significantly correlated with a more advanced T stage (T2 versus T1) (*P* = .004), positive lymph nodes (*P* < .001), the presence of tumor necrosis (*P* = .016), and greater tumour thickness (*P* < .001). Interestingly, the patients expressing OPN showed a significantly lower DFS rate (63.4% versus 92.8%; log-rank test, *P* < .001).

Using the method developed by Matsuzaki et al. [130], OPN expression still significantly related to the expression of VEGF and CD105 (both *P* < .001), tumour invasion depth (*P* = .001), and regional nodal metastases (*P* < .001), and Chien et al. [131] confirmed the relationship between OPN and VEGF, thus suggesting their importance in the development of new vessels in early tongue cancer. Hypoxia can also contribute to the increased expression of OPN and the activation of other important angiogenetic factors. These data argue in favour of a role of OPN in predicting a poor prognosis, and, therefore, possibly in influencing the decision to adopt more aggressive therapy.

## 9. Survivin: OSCC (c)

A large number of cancer cells acquire resistance to treatment by evading apoptosis, and a family of inhibitors of apoptosis proteins (IAPs) can interfere with programmed cell death [132, 133]. The ultimate effectors of the apoptotic machinery are the intracellular proteases called caspases [134]. Caspase-8 and -9 trigger the activation of more caspases that execute the cell death program. It is believed that survivin, which is encoded by the gene BIRC5, blocks caspase-mediated death by forming a stable complex with X-IAP, which has a synergistic inhibitory action on apoptosis [135]. Survivin is highly expressed in many cancer types and has been associated with a more aggressive phenotype and poor outcome in oral SCC [136]. In their study of OSCC in Taiwan, Lin et al. found no significant correlation between survivin expression and patient age, gender, oral habits, cancer location, or TNM status, but the patients with high survivin expression, an advanced stage, a larger tumour size or positive lymph node metastases had a significantly shorter OS than the others (*P* = .014, .012, .005, and .011, log-rank test) [137]. Survivin protein expression may thus be considered an important early event in oral carcinogenesis and predicts an unfavourable prognosis for OSCC. On the other hand, Freier et al. found no statistical difference between tumours with a gain in *BIRC5* gene copy number and those with a balanced *BIRC5* locus (*P* > .05) in terms of the prevalence of high survivin expression, and high survivin expression predicted longer OS in a subgroup of patients with advanced tumours treated by radiotherapy [138]. The authors concluded that the additional *BIRC5* copies were probably biologically inactive, that another distinct molecular mechanism might be responsible for high survivin expression in OSCC, and that survivin might be used to define better the patients who may benefit from radiation therapy. 

The difference in these results may also have been due to the evaluation system used, because the latter study used a score that took into account both nuclear and cytoplasmic cells. A study of nuclear staining alone in breast and colon cancer found a correlation with better survival, and so it is reasonable to imagine that Freier's finding of an impact on survival was due to the nuclear expression of survivin. 

Unfortunately all of these studies involved OSCC series that included only a minority of patients affected by tongue cancer. In our own recent series of tongue cancer patients, immunohistochemical analysis of survivin did not correlate with DFS or OS (unpublished data).

## 10. EGFR

Epidermal growth factor receptor (EGFR) is a 170-kDa transmembrane glycoprotein whose gene is located on chromosome 7p12. It is a member of the family of tyrosine kinase (TK) growth factor receptors, a group of proteins whose aberrant activity plays a key role in cell growth and neoplastic progression [139, 140]. A number of extracellular growth factor ligands, including epidermal growth factor (EGF) and transforming growth factor alpha (TGF-*α*), bind to EGFR and thus lead to the downstream activation of ras, which ultimately leads to cell cycle progression, decreased apoptosis, as well as increased angiogenesis and metastatic properties [141, 142]. EGFR and its ligand TGF-*α* are overexpressed in nearly all HNC [143, 144], and its expression is typically associated with greater radio- chemoresistance and shorter DFS and OS [145, 146]. EGFR expression has been reported to be 29% and 50% in hypopharyngeal and oral cavity cancers [147], and ranging from 42% to 80% in other types of HNC [148, 149]. 

Few data are available concerning the expression and prognostic value of EGFR in tongue cancer. EGFR mutations are not frequent (they have been found in 14% of investigated cases) and, unlike in nonsmall cell lung cancer (NSCLC), they do not correlate with prognosis [150]. Treatment with tyrosine kinase inhibitors is less effective in HNC than NSCLC [151, 152] although the types of mutations are very similar [153]. Mahmoud et al. studied HNC specimens for EGFR mutations and expression in a Japanese population and found a silent mutation in only one case, thus reflecting the low incidence reported in previous studies, whereas overexpression (+2, +3) was found in 68% of the tumours. EGFR overexpression was significantly associated with poor tumor differentiation (*P* = .02) and a positive nodal stage (*P* = .032) [154].

As in the case of Western patients, mutations are rare in Japanese HNC [155, 156], and protein overexpression rather than mutation might be responsible for activating the EGFR pathway. Ulanovski et al. studied 27 patients who underwent surgery for SCC of the tongue. EGFR and erb-B2 were expressed in 34% and 17% of the specimens, but the authors could not demonstrate any association between EGFR expression or erbB2, and tumour depth, lymph node status, extracapsular invasion, recurrence, or survival [157].

## 11. Conclusions

Cancer of the tongue is frequent and has a poor prognosis, with a 5-year survival rate of less than 50%. Treatments should be individualized on the basis of the biological characteristics of the tumour with the aim of improving locoregional control, preventing distant metastases, and lengthening survival. The role of EBV and HPV is very slight, although the latter may indicate a better prognosis. Among hypoxia markers, only the expression of EPOR and pimonidazole correlates with locoregional control and DFS, but these findings are based on a small number of patients. VEGF, tight junction proteins, and p53 expression hardly correlate with poor prognostic features, and the survivin findings are also controversial although it may be useful to select a subpopulation of patients who may benefit from radiation therapy. The intracytoplasmic expression of erbB2 and the ki-67 proliferation index are associated with OS, and FAS expression is related to aggressive histological features. OPN is a VEGF-inducible factor, that is, overexpressed in cases of aggressive cell behaviour, and is associated with decreased DFS, at least in T1-2 tumours. EGFR mutations are seldom found in tongue cancer and do not play a significant prognostic role; likewise, EGFR overexpression correlates with nodal stage but not DFS or OS. 

In this era of targeted therapies tailored on the biological characteristics of tumours, the results, as far as tongue cancer is concerned, are still poor and conflicting, and new insights are eagerly expected with the aim of offering the best possible treatment to each patient. 

Antiangiogenetics, anti-EGFR, tyrosine-kinase inhibitors and proapoptotics are all factors deserving further evaluation in order to improve outcomes in patients affected by cancer of the tongue.

## References

[1] Pimenta Amaral TM, Da Silva F, Carvalho AL, Pinto CAL, Kowalski LP (2004). Predictive factors of occult metastasis and prognosis of clinical stages I and II squamous cell carcinoma of the tongue and floor of the mouth. *Oral Oncology*.

[2] Kademani D, Bell RB, Bagheri S (2005). Prognostic factors in intraoral squamous cell carcinoma: the influence of histologic grade. *Journal of Oral and Maxillofacial Surgery*.

[3] Shantz SP, Harrison LB, Forastiere AA, De Vita T, Hellman S, Rosemberg SA Tumors of the nasal cavity and paranasal sinuses, nasopharynx, oral cavity, and oropharynx. *Principles and Practice of Oncology*.

[4] Byers RM (1975). Squamous cell carcinoma of the oral tongue in patients less than thirty years of age. *American Journal of Surgery*.

[5] Jones JB, Lampe HB, Cheung HW (1989). Carcinoma of the tongue in young patients. *Journal of Otolaryngology*.

[6] Llewellyn CD, Johnson NW, Warnakulasuriya KA (2001). Risk factors for squamous cell carcinoma of the oral cavity in young people—a comprehensive literature review. *Oral Oncology*.

[7] Shiboski CH, Schmidt BL, Jordan RCK (2005). Tongue and tonsil carcinoma: increasing trends in the U.S. population ages 20–44 years. *Cancer*.

[8] Annertz K, Anderson H, Biörklund A (2002). Incidence and survival of squamous cell carcinoma of the tongue in Scandinavia, with special reference to young adults. *International Journal of Cancer*.

[9] Depue RH (1986). Rising mortality from cancer of the tongue in young white males. *The New England Journal of Medicine*.

[10] Sarkaria JN, Harari PM (1994). Oral tongue cancer in young adults less than 40 years of age: rationale for aggressive therapy. *Head and Neck*.

[11] Byers RM (1975). Squamous cell carcinoma of the oral tongue in patients less than thirty years of age. *American Journal of Surgery*.

[12] Zhen W, Karnell LH, Hoffman HT, Funk GF, Buatti JM, Menck HR (2004). The national cancer data base report on squamous cell carcinoma of the base of tongue. *Head and Neck*.

[13] Mashberg A, Boffetta P, Winkelman R, Garfinkel L (1993). Tobacco smoking, alcohol drinking, and cancer of the oral cavity and oropharynx among U.S. Veterans. *Cancer*.

[14] Boffetta P, Mashberg A, Winkelmann R, Garfinkel L (1992). Carcinogenic effect of tobacco smoking and alcohol drinking on anatomic sites of the oral cavity and oropharynx. *International Journal of Cancer*.

[15] Gebhart E, Liehr T (2000). Patterns of genomic imbalances in human solid tumors (Review). *International Journal of Oncology*.

[16] Brzoska PM, Levin NA, Fu KK (1995). Frequent novel DNA copy number increase in squamous cell head and neck tumors. *Cancer Research*.

[17] Bergamo NA, Rogatto SR, Poli-Frederico RC (2000). Comparative genomic hybridization analysis detects frequent over-representation of DNA sequences at 3q, 7p, and 8q in head and neck carcinomas. *Cancer Genetics and Cytogenetics*.

[18] Singh B, Gogineni SK, Sacks PG (2001). Molecular cytogenetic characterization of head and neck squamous cell carcinoma and refinement of 3q amplification. *Cancer Research*.

[19] Speicher MR, Howe C, Crotty P, du Manoir S, Costa J, Ward DC (1995). Comparative genomic hybridization detects novel deletions and amplifications in head and neck squamous cell carcinomas. *Cancer Research*.

[20] Bockmuhl U, Wolf G, Schmidt S (1998). Genomic alterations associated with malignancy in head and neck cancer. *Head and Neck*.

[21] Weber RG, Scheer M, Born IA (1998). Recurrent chromosomal imbalances detected in biopsy material from oral premalignant and malignant lesions by combined tissue microdissection, universal DNA amplification, and comparative genomic hybridization. *American Journal of Pathology*.

[22] Singh B, Stoffel A, Gogineni S (2002). Amplification of the 3q26.3 locus is associated with progression to invasive cancer and is a negative prognostic factor in head and neck squamous cell carcinomas. *American Journal of Pathology*.

[23] Redon R, Muller D, Caulee K, Wanherdrick K, Abecassis J, du Manoir S (2001). A simple specific pattern of chromosomal aberrations at early stages of head and neck squamous cell carcinomas: *PIK3CA* but not *p63* gene as a likely target of 3q26-qter gains. *Cancer Research*.

[24] Al Moustafa A-E, Alaoui-Jamali MA, Batist G (2002). Identification of genes associated with head and neck carcinogenesis by cDNA microarray comparison between matched primary normal epithelial and squamous carcinoma cells. *Oncogene*.

[25] Pattle SB, Farrell PJ (2006). The role of Epstein-Barr virus in cancer. *Expert Opinion on Biological Therapy*.

[26] Young LS, Murray PG (2003). Epstein-Barr virus and oncogenesis: from latent genes to tumours. *Oncogene*.

[27] Liebowitz D (1994). Nasopharyngeal carcinoma: the Epstein-Barr virus association. *Seminars in Oncology*.

[28] Borza CM, Hutt-Fletcher LM (2002). Alternate replication in B cells and epithelial cells switches tropism of Epstein-Barr virus. *Nature Medicine*.

[29] Niedobitek G, Young LS (1994). Epstein-Barr virus persistence and virus-associated tumours. *The Lancet*.

[30] Pathmanathan R, Prasad U, Sadler R, Flynn K, Raab-Traub N (1995). Clonal proliferations of cells infected with Epstein-Barr virus in preinvasive lesions related to nasopharyngeal carcinoma. *The New England Journal of Medicine*.

[31] Frangou P, Buettner M, Niedobitek G (2005). Epstein-Barr virus (EBV) infection in epithelial cells in vivo: rare detection of EBV replication in tongue mucosa but not in salivary glands. *Journal of Infectious Diseases*.

[32] Herrero R, Castellsagué X, Pawlita M (2003). Human papillomavirus and oral cancer: the international agency for research on cancer multicenter study. *Journal of the National Cancer Institute*.

[33] Mork J, Lie AK, Glattre E (2001). Human papillomavirus infection as a risk factor for squamous-cell carcinoma of the head and neck. *The New England Journal of Medicine*.

[34] Gillison ML, Shah KV (2001). Human papillomavirus-associated head and neck squamous cell carcinoma: mounting evidence for an etiologic role for human papillomavirus in a subset of head and neck cancers. *Current Opinion in Oncology*.

[35] Sugerman PB, Shillitoe EJ (1997). The high risk human papillomaviruses and oral cancer: evidence for and against a causal relationship. *Oral Diseases*.

[36] Koskinen WJ, Chen RW, Leivo I (2003). Prevalence and physical status of human papillomavirus in squamous cell carcinomas of the head and neck. *International Journal of Cancer*.

[37] Kantola S, Parikka M, Jokinen K (2000). Prognostic factors in tongue cancer—relative importance of demographic, clinical and histopathological factors. *British Journal of Cancer*.

[38] Dahlgren L, Dahlstrand H, Lindquist D (2004). Human papillomavirus is more common in base of tongue than in mobile tongue cancer and is a favorable prognostic factor in base of tongue cancer patients. *International Journal of Cancer*.

[39] Liang X-H, Lewis J, Foote R, Smith D, Kademani D (2008). Prevalence and significance of human papillomavirus in oral tongue cancer: the Mayo Clinic experience. *Journal of Oral and Maxillofacial Surgery*.

[40] Brizel DM, Sibley GS, Prosnitz LR, Scher RL, Dewhirst MW (1997). Tumor hypoxia adversely affects the prognosis of carcinoma of the head and neck. *International Journal of Radiation Oncology Biology Physics*.

[41] Jonathan RA, Wijffels KIEM, Peeters W (2006). The prognostic value of endogenous hypoxia-related markers for head and neck squamous cell carcinomas treated with ARCON. *Radiotherapy and Oncology*.

[42] Durand RE (1994). The influence of microenvironmental factors during cancer therapy. *In Vivo*.

[43] Olive PL, Durand RE (1994). Drug and radiation resistance in spheroids: cell contact and kinetics. *Cancer and Metastasis Reviews*.

[44] Teicher BA (1994). Hypoxia and drug resistance. *Cancer and Metastasis Reviews*.

[45] Janssen HL, Haustermans KM, Balm AJ, Begg AC (2005). Hypoxia in head and neck cancer: how much, how important?. *Head and Neck*.

[46] Dachs GU, Chaplin DJ (1998). Microenvironmental control of gene expression: implications for tumor angiogenesis, progression, and metastasis. *Seminars in Radiation Oncology*.

[47] Dachs GU, Tozer GM (2000). Hypoxia modulated gene expression: angiogenesis, metastasis and therapeutic exploitation. *European Journal of Cancer*.

[48] De Jaeger K, Kavanagh M-C, Hill RP (2001). Relationship of hypoxia to metastatic ability in rodent tumours. *British Journal of Cancer*.

[49] Beasley NJP, Wykoff CC, Watson PH (2001). Carbonic anhydrase IX, an endogenous hypoxia marker, expression in head and neck squamous cell carcinoma and its relationship to hypoxia, necrosis, and microvessel density. *Cancer Research*.

[50] Hui EP, Chan ATC, Pezzella F (2002). Coexpression of hypoxia-inducible factors 1*α* and 2*α*, carbonic anhydrase IX, and vascular endothelial growth factor in nasopharyngeal carcinoma and relationship to survival. *Clinical Cancer Research*.

[51] Roh J-L, Cho K-J, Kwon GY (2009). The prognostic value of hypoxia markers in T2-staged oral tongue cancer. *Oral Oncology*.

[52] Kennedy AS, Raleigh JA, Perez GM (1997). Proliferation and hypoxia in human squamous cell carcinoma of the cervix: first report of combined immunohistochemical assays. *International Journal of Radiation Oncology Biology Physics*.

[53] Varia MA, Calkins-Adams DP, Rinker LH (1998). Pimonidazole: a novel hypoxia marker for complementary study of tumor hypoxia and cell proliferation in cervical carcinoma. *Gynecologic Oncology*.

[54] Wijffels KI, Kaanders JH, Rijken PF (2000). Vascular architecture and hypoxic profiles in human head and neck squamous cell carcinomas. *British Journal of Cancer*.

[55] Kaanders JH, Wijffels KI, Marres HAM (2002). Pimonidazole binding and tumor vascularity predict for treatment outcome in head and neck cancer. *Cancer Research*.

[56] Veikkola T, Karkkainen M, Claesson-Welsh L, Alitalo K (2000). Regulation of angiogenesis via vascular endothelial growth factor receptors. *Cancer Research*.

[57] Ferrara N, Davis-Smyth T (1997). The biology of vascular endothelial growth factor. *Endocrine Reviews*.

[58] Kukk E, Lymboussaki A, Taira S (1996). VEGF-C receptor binding and pattern of expression with VEGFR-3 suggests a role in lymphatic vascular development. *Development*.

[59] Roskoski R (2007). Vascular endothelial growth factor (VEGF) signaling in tumor progression. *Critical Reviews in Oncology/Hematology*.

[60] Cohen T, Gitay-Goren H, Sharon R (1995). VEGF121, a vascular endothelial growth factor (VEGF) isoform lacking heparin binding ability, requires cell-surface heparan sulfates for efficient binding to the VEGF receptors of human melanoma cells. *The Journal of Biological Chemistry*.

[61] Salven P, Lymboussaki A, Heikkila P (1998). Vascular endothelial growth factors VEGF-B and VEGF-C are expressed in human tumors. *American Journal of Pathology*.

[62] Jeltsch M, Kaipainen A, Joukov V (1997). Hyperplasia of lymphatic vessels in VEGF-C transgenic mice. *Science*.

[63] Oh S-J, Jeltsch MM, Birkenhager R (1997). VEGF and VEGF-C: specific induction of angiogenesis and lymphangiogenesis in the differentiated avian chorioallantoic membrane. *Developmental Biology*.

[64] Kaipainen A, Korhonen J, Mustonen T (1995). Expression of the fms-like tyrosine kinase 4 gene becomes restricted to lymphatic endothelium during development. *Proceedings of the National Academy of Sciences of the United States of America*.

[65] Kukk E, Lymboussaki A, Taira S (1996). VEGF-C receptor binding and pattern of expression with VEGFR-3 suggests a role in lymphatic vascular development. *Development*.

[66] Benefield J, Petruzzelli GJ, Fowler S, Taitz A, Kalkanis J, Young MRI (1996). Regulation of the steps of angiogenesis by human head and neck squamous cell carcinomas. *Invasion and Metastasis*.

[67] O-Charoenrat P, Rhys-Evans P, Eccles SA (2001). Expression of vascular endothelial growth factor family members in head and neck squamous cell carcinoma correlates with lymph node metastasis. *Cancer*.

[68] Siriwardena BS, Kudo Y, Ogawa I, Udagama MN, Tilakaratne WM, Takata T (2008). VEGF-C is associated with lymphatic status and invasion in oral cancer. *Journal of Clinical Pathology*.

[69] Kishimoto K, Sasaki A, Yoshihama Y, Mese H, Tsukamoto G, Matsumura T (2003). Expression of vascular endothelial growth factor-C predicts regional lymph node metastasis in early oral squamous cell carcinoma. *Oral Oncology*.

[70] Kim SH, Cho NH, Kim K (2006). Correlations of oral tongue cancer invasion with matrix metalloproteinases (MMPs) and vascular endothelial growth factor (VEGF) expression. *Journal of Surgical Oncology*.

[71] Faustino SES, Oliveira DT, Nonogaki S, Landman G, Carvalho AL, Kowalski LP (2008). Expression of vascular endothelial growth factor-C does not predict occult lymph-node metastasis in early oral squamous cell carcinoma. *International Journal of Oral and Maxillofacial Surgery*.

[72] Cho JH, Kim H-S, Park C-S (2007). Maspin expression in early oral tongue cancer and its relation to expression of mutant-type p53 and vascular endothelial growth factor (VEGF). *Oral Oncology*.

[73] Sheng S (2004). The promise and challenge toward the clinical application of maspin in cancer. *Frontiers in Bioscience*.

[74] Zhang M, Volpert O, Shi YH, Bouck N (2000). Maspin is an angiogenesis inhibitor. *Nature Medicine*.

[75] Le Querrec A, Duval D, Tobelem G (1993). Tumour angiogenesis. *Bailliere's Clinical Haematology*.

[76] Ellerbroek SM, Halbleib JM, Benavidez M (2001). Phosphatidylinositol 3-kinase activity in epidermal growth factor-stimulated matrix metalloproteinase-9 production and cell surface association. *Cancer Research*.

[77] Agarwal R, D'Souza T, Morin PJ (2005). Claudin-3 and claudin-4 expression in ovarian epithelial cells enhances invasion and is associated with increased matrix metalloproteinase-2 activity. *Cancer Research*.

[78] Hayashida Y, Honda K, Idogawa M (2005). E-cadherin regulates the association between *β*-catenin and actinin-4. *Cancer Research*.

[79] Nei H, Saito T, Tobioka H, Itoh E, Mori M, Kudo R (1996). Expression of component desmosomal proteins in uterine endometrial carcinoma and their relation to cellular differentiation. *Cancer*.

[80] Bazarbachi A, Abou Merhi R, Gessain A (2004). Human T-cell lymphotropic virus type I-infected cells extravasate through the endothelial barrier by a local angiogenesis-like mechanism. *Cancer Research*.

[81] Gonzalez-Mariscal L, Betanzos A, Nava P, Jaramillo BE (2003). Tight junction proteins. *Progress in Biophysics and Molecular Biology*.

[82] Mitic LL, Anderson JM (1998). Molecular architecture of tight junctions. *Annual Review of Physiology*.

[83] Mullin JM (1997). Potential interplay between luminal growth factors and increased tight junction permeability in epithelial carcinogenesis. *Journal of Experimental Zoology*.

[84] Soini Y (2005). Expression of claudins 1, 2, 3, 4, 5 and 7 in various types of tumours. *Histopathology*.

[85] Oku N, Sasabe E, Ueta E, Yamamoto T, Osaki T (2006). Tight junction protein claudin-1 enhances the invasive activity of oral squamous cell carcinoma cells by promoting cleavage of laminin-5 *γ*2 chain via matrix metalloproteinase (MMP)-2 and membrane-type MMP-1. *Cancer Research*.

[86] Bello IO, Vilen S-T, Niinimaa A, Kantola S, Soini Y, Salo T (2008). Expression of claudins 1, 4, 5, and 7 and occludin, and relationship with prognosis in squamous cell carcinoma of the tongue. *Human Pathology*.

[87] Popescu NC, King CR, Kraus MH (1989). Localization of the human ErbB-2 gene on normal and rearranged chromosomes 17 to bands q12-21.32. *Genomics*.

[88] Olayioye MA, Neve RM, Lane HA, Hynes NE (2000). The ErbB signaling network: receptor heterodimerization in development and cancer. *The EMBO Journal*.

[89] Xia W, Lau Y-K, Zhang H-Z (1997). Strong correlation between c-ErbB-2 overexpression and overall survival of patients with oral squamous cell carcinoma. *Clinical Cancer Research*.

[90] Xia W, Lau Y-K, Zhang H-Z (1999). Combination of EGFR, HER-2/neu, and HER-3 is a stronger predictor for the outcome of oral squamous cell carcinoma than any individual family members. *Clinical Cancer Research*.

[91] Werkmeister R, Brandt B, Joos U (2000). Clinical relevance of ErbB-1 and -2 oncogenes in oral carcinomas. *Oral Oncology*.

[92] Bei R, Pompa G, Vitolo D (2001). Co-localization of multiple ErbB receptors in stratified epithelium of oral squamous cell carcinoma. *Journal of Pathology*.

[93] Khan AJ, King BL, Smith BD (2002). Characterization of the HER-2/neu oncogene by immunohistochemical and fluorescence in situ hybridization analysis in oral and oropharyngeal squamous cell carcinoma. *Clinical Cancer Research*.

[94] Khademi B, Shirazi FM, Vasei M (2002). The expression of p53, c-ErbB-1 and c-ErbB-2 molecules and their correlation with prognostic markers in patients with head and neck tumors. *Cancer Letters*.

[95] Innocenzi D, Alo PL, Balzani A (2003). Fatty acid synthase expression in melanoma. *Journal of Cutaneous Pathology*.

[96] Takahiro T, Shinichi K, Toshimitsu S (2003). Expression of fatty acid synthase as a prognostic indicator in soft tissue sarcomas. *Clinical Cancer Research*.

[97] Alo PL, Visca P, Framarino ML (2000). Immunohistochemical study of fatty acid synthase in ovarian neoplasms. *Oncology Reports*.

[98] Kusakabe T, Nashimoto A, Honma K, Suzuki T (2002). Fatty acid synthase is highly expressed in carcinoma, adenoma and in regenerative epithelium and intestinal metaplasia of the stomach. *Histopathology*.

[99] Silva SD, Agostini M, Nishimoto IN (2004). Expression of fatty acid synthase, ErbB2 and Ki-67 in head and neck squamous cell carcinoma. A clinicopathological study. *Oral Oncology*.

[100] Agostini M, Silva SD, Zecchin KG (2004). Fatty acid synthase is required for the proliferation of human oral squamous carcinoma cells. *Oral Oncology*.

[101] Jayakumar A, Tai M-H, Huang W-Y (1995). Human fatty acid synthase: properties and molecular cloning. *Proceedings of the National Academy of Sciences of the United States of America*.

[102] Menendez JA, Lupu R, Colomer R (2005). Targeting fatty acid synthase: potential for therapeutic intervention in Her-2/neu-overexpressing breast cancer. *Drug News and Perspectives*.

[103] Silva SD, Perez DE, Alves FA (2008). ErbB2 and fatty acid synthase (FAS) expression in 102 squamous cell carcinomas of the tongue: correlation with clinical outcomes. *Oral Oncology*.

[104] Silva SD, Perez DE, Nishimoto IN (2008). Fatty acid synthase expression in squamous cell carcinoma of the tongue: clinicopathological findings. *Oral Diseases*.

[105] Angiero F, Sordo RD, Dessy E (2008). Comparative analysis of c-ErbB-2 (HER-2/neu) in squamous cell carcinoma of the tongue: does over-expression exist? And what is its correlation with traditional diagnostic parameters?. *Journal of Oral Pathology and Medicine*.

[106] Chang F, Syrjanen S, Syrjanen K (1995). Implications of the p53 tumor-suppressor gene in clinical oncology. *Journal of Clinical Oncology*.

[107] Lowe SW, Bodis S, McClatchey A (1994). p53 status and the efficacy of cancer therapy in vivo. *Science*.

[108] Lowe SW, Ruley HE, Jacks T, Housman DE (1993). p53-dependent apoptosis modulates the cytotoxicity of anticancer agents. *Cell*.

[109] Suzuki H, Takahashi T, Kuroishi T (1992). p53 mutations in non-small cell lung cancer in Japan: association between mutations and smoking. *Cancer Research*.

[110] Wang X, Christiani DC, Wiencke JK (1995). Mutations in the p53 gene in lung cancer are associated with cigarette smoking and asbestos exposure. *Cancer Epidemiology Biomarkers and Prevention*.

[111] Field JK, Spandidos DA, Malliri A, Gosney JR, Yiagnisis M, Stell PM (1991). Elevated P53 expression correlates with a history of heavy smoking in squamous cell carcinoma of the head and neck. *British Journal of Cancer*.

[112] Matthews JB, Scully C, Jovanovic A, Van der Waal I, Yeudall WA, Prime SS (1993). Relationship of tobacco/alcohol use to p53 expression in patients with lingual squamous cell carcinomas. *European Journal of Cancer Part B*.

[113] Atula S, Kurvinen K, Grinman R, Syrjken S (1996). SSCP pattern indicative for p53 mutation is related to advanced stage and high-grade of tongue cancer. *European Journal of Cancer Part B*.

[114] Sameshima Y, Matsuno Y, Hirohashi S (1992). Alterations of the p53 gene are common and critical events for the maintenance of malignant phenotypes in small-cell lung carcinoma. *Oncogene*.

[115] Pohl J, Goldfinger N, Radler-Pohl A, Rotter V, Schirrmacher V (1988). p53 increases experimental metastatic capacity of murine carcinoma cells. *Molecular and Cellular Biology*.

[116] Vogelstein B (1990). A deadly inheritance. *Nature*.

[117] Vora HH, Trivedi TI, Shukla SN, Shah NG, Goswami JV, Shah PM (2006). p53 expression in leukoplakia and carcinoma of the tongue. *International Journal of Biological Markers*.

[118] Nagler RM, Kerner H, Laufer D, Ben-Eliezer S, Minkov I, Ben-Itzhak O (2002). Squamous cell carcinoma of the tongue: the prevalence and prognostic roles of p53, Bcl-2, c-ErbB-2 and apoptotic rate as related to clinical and pathological characteristics in a retrospective study. *Cancer Letters*.

[119] Keum KC, Chung EJ, Koom WS (2006). Predictive value of p53 and PCNA expression for occult neck metastases in patients with clinically node-negative oral tongue cancer. *Otolaryngology: Head and Neck Surgery*.

[120] Senger DR, Wirth DF, Hynes RO (1979). Transformed mammalian cells secrete specific proteins and phosphoproteins. *Cell*.

[121] Liaw L, Skinner MP, Raines EW (1995). The adhesive and migratory effects of osteopontin are mediated via distinct cell surface integrins. Role of *α*(v)*β*3 in smooth muscle cell migration to osteopontin in vitro. *Journal of Clinical Investigation*.

[122] Wai PY, Kuo PC (2004). The role of osteopontin in tumor metastasis. *Journal of Surgical Research*.

[123] Singhai H, Bautista DS, Tonkin KS (1997). Elevated plasma osteopontin in metastatic breast cancer associated with increased tumor burden and decreased survival. *Clinical Cancer Research*.

[124] Ue T, Yokozaki H, Kitadai Y (1998). Co-expression of osteopontin and CD44v9 in gastric cancer. *International Journal of Cancer*.

[125] Kim J-H, Skates SJ, Uede T (2002). Osteopontin as a potential diagnostic biomarker for ovarian cancer. *Journal of the American Medical Association*.

[126] Agrawal D, Chen T, Irby R (2002). Osteopontin identified as lead marker of colon cancer progression, using pooled sample expression profiling. *Journal of the National Cancer Institute*.

[127] Oates AJ, Barraclough R, Rudland PS (1997). The role of osteopontin in tumorigenesis and metastasis. *Invasion and Metastasis*.

[128] Weber GF (2001). The metastasis gene osteopontin: a candidate target for cancer therapy. *Biochimica et Biophysica Acta*.

[129] Tuck AB, Arsenault DM, O'Malley FP (1999). Osteopontin induces increased invasiveness and plasminogen activator expression of human mammary epithelial cells. *Oncogene*.

[130] Matsuzaki H, Shima K, Muramatsu T (2007). Osteopontin as biomarker in early invasion by squamous cell carcinoma in tongue. *Journal of Oral Pathology and Medicine*.

[131] Chien C-Y, Su C-Y, Chuang H-C (2008). Clinical significance of osteopontin expression in T1 and T2 tongue cancers. *Head and Neck*.

[132] Ambrosini G, Adida C, Altieri DC (1997). A novel anti-apoptosis gene, survivin, expressed in cancer and lymphoma. *Nature Medicine*.

[133] Deveraux QL, Reed JC (1999). IAP family proteins—suppressors of apoptosis. *Genes and Development*.

[134] Thornberry NA (1999). Caspases: a decade of death research. *Cell Death and Differentiation*.

[135] Dohi T, Okada K, Xia F (2004). An IAP-IAP complex inhibits apoptosis. *The Journal of Biological Chemistry*.

[136] Lo Muzio L, Farina A, Rubini C (2005). Survivin as prognostic factor in squamous cell carcinoma of the oral cavity. *Cancer Letters*.

[137] Lin C-Y, Hung H-C, Kuo R-C, Chiang C-P, Kuo MY-P (2005). Survivin expression predicts poorer prognosis in patients with areca quid chewing-related oral squamous cell carcinoma in Taiwan. *Oral Oncology*.

[138] Freier K, Pungs S, Sticht C (2007). High survivin expression is associated with favorable outcome in advanced primary oral squamous cell carcinoma after radiation therapy. *International Journal of Cancer*.

[139] Carpenter G, Cohen S (1990). Epidermal growth factor. *The Journal of Biological Chemistry*.

[140] Hardisson D (2003). Molecular pathogenesis of head and neck squamous cell carcinoma. *European Archives of Oto-Rhino-Laryngology*.

[141] Kalyankrishna S, Grandis JR (2006). Epidermal growth factor receptor biology in head and neck cancer. *Journal of Clinical Oncology*.

[142] Grandis JR, Tweardy DJ (1993). Elevated levels of transforming growth factor and epidermal growth factor receptor messenger RNA are early markers of carcinogenesis in head and neck cancer. *Cancer Research*.

[143] Pivot X, Magné N, Guardiola E (2005). Prognostic impact of the epidermal growth factor receptor levels for patients with larynx and hypopharynx cancer. *Oral Oncology*.

[144] Hitt R, Ciruelos E, Amador ML (2005). Prognostic value of the epidermal growth factor receptor (EGRF) and p53 in advanced head and neck squamous cell carcinoma patients treated with induction chemotherapy. *European Journal of Cancer*.

[145] Ang KK, Berkey BA, Tu X (2002). Impact of epidermal growth factor receptor expression on survival and pattern of relapse in patients with advanced head and neck carcinoma. *Cancer Research*.

[146] Liang K, Ang KK, Milas L, Hunter N, Fan Z (2003). The epidermal growth factor receptor mediates radioresistance. *International Journal of Radiation Oncology Biology Physics*.

[147] Katoh K, Nakanishi Y, Akimoto S (2002). Correlation between laminin-5 *γ*2 chain expression and epidermal growth factor receptor expression and its clinicopathological significance in squamous cell carcinoma of the tongue. *Oncology*.

[148] Santini J, Formento J-L, Francoual M (1991). Characterization, quantification, and potential clinical value of the epidermal growth factor receptor in head and neck squamous cell carcinomas. *Head and Neck*.

[149] Grandis JR, Melhem MF, Barnes EL, Tweardy DJ (1996). Quantitative immunohistochemical analysis of transforming growth factor-*α* and epidermal growth factor receptor in patients with squamous cell carcinoma of the head and neck. *Cancer*.

[150] Eberhard DA, Johnson BE, Amler LC (2005). Mutations in the epidermal growth factor receptor and in KRAS are predictive and prognostic indicators in patients with non-small-cell lung cancer treated with chemotherapy alone and in combination with erlotinib. *Journal of Clinical Oncology*.

[151] Wirth LJ, Haddad RI, Lindeman NI (2005). Phase I study of gefitinib plus celecoxib in recurrent or metastatic squamous cell carcinoma of the head and neck. *Journal of Clinical Oncology*.

[152] Cohen EEW, Rosen F, Stadler WM (2003). Phase II trial of ZD1839 in recurrent or metastatic squamous cell carcinoma of the head and neck. *Journal of Clinical Oncology*.

[153] Lee JW, Soung YH, Kim SY (2005). Somatic mutations of *EGFR* gene in squamous cell carcinoma of the head and neck. *Clinical Cancer Research*.

[154] Mahmoud AL, Sheikh A, Gunduz GM (2008). Expression and mutation analysis of epidermal growth factor receptor in head and neck squamous cell carcinoma. *Cancer Science*.

[155] Lemos-González Y, Páez de la Cadena M, Rodríguez-Berrocal FJ, Rodríguez-Piñeiro AM, Pallas E, Valverde D (2007). Absence of activating mutations in the EGFR kinase domain in spanish head and neck cancer patients. *Tumor Biology*.

[156] Loeffler-Ragg J, Witsch-Baumgartner M, Tzankov A (2006). Low incidence of mutations in EGFR kinase domain in Caucasian patients with head and neck squamous cell carcinoma. *European Journal of Cancer*.

[157] Ulanovski D, Stern Y, Roizman P, Shpitzer T, Popovtzer A, Feinmesser R (2004). Expression of EGFR and Cerb-B2 as prognostic factors in cancer of the tongue. *Oral Oncology*.

